# Correlations between life course trauma on perinatal outcomes of women on community supervision – a retrospective cross sectional study

**DOI:** 10.1186/s12884-026-09495-2

**Published:** 2026-09-03

**Authors:** Allison D. Ihle, Karenna Thomas, Breann Wicks, Min Wang, Laura B. Sisk, Jacqueline M. McGrath

**Affiliations:** 1 University of Texas at Austin School of Nursing, Austin, USA; 2 Department of Pediatrics, Medical School, University of Minnesota, 717 Delaware Street SE, Minneapolis, MN 55414 USA; 3 Department of Statistics and Data Science, The University of Texas at San Antonio, One UTSA Circle, San Antonio, TX 78249 USA; 4 School of Nursing, The University of Texas Health at San Antonio, 7703 Floyd Curl Drive, San Antonio, TX 78229 USA

**Keywords:** Community supervision, Criminal legal system, Perinatal health, Birthing people, Women, Life course, Trauma, ACEs

## Abstract

**Background:**

Women on probation (community supervision) report high rates of adverse childhood experiences (ACEs) and ongoing trauma, yet little research has examined how trauma across the life course relates to perinatal outcomes during community supervision. This study examined associations between lifetime trauma and perinatal violence, infant separation, and postpartum mood symptoms among women who experienced pregnancy, childbirth, and postpartum while on probation.

**Methods:**

We conducted a cross-sectional secondary analysis from N = 60 women in South-Central Texas who had a pregnancy within the past five years while on community supervision. Trauma exposures included selected ACEs and adulthood/perinatal trauma (domestic, intimate partner, and sexual violence). Outcomes were perinatal violence, permanent infant removal after childbirth, and postpartum mood symptoms (PHQ-2/GAD-2). We used logistic regression to examine associations between trauma exposures and outcomes; given the ordinal structure of key trauma measures, we used Kendall’s tau to estimate correlations between cumulative ACEs/lifetime trauma and perinatal outcomes.

**Results:**

Participants spent an average of 5 years (SD = 3) on community supervision, and most reported partner violence (*n* = 52, 87%); no mental health care in the year prior to their last arrest (*n* = 55, 92%); and possible major depressive disorder (*n* = 20, 33%) and generalized anxiety disorder (*n* = 34, 57%). ACE prevalence included caregiver incarceration (*n* = 34, 56%), physical abuse (*n* = 25, 42%), witnessing abuse (*n* = 27, 45%), foster care (*n* = 17, 8%), and childhood sexual abuse (*n* = 29, 48%); 25% reported four (*n* = 9, 16%) or five (*n* = 6, 10%) ACEs. Cumulative ACE exposure was correlated with perinatal violence (*p* = 0.03), and childhood forced sexual intercourse was associated with perinatal violence (*p* = 0.04). Trauma in adulthood was associated with permanent infant removal (*p* = 0.003). Sexual violence in adulthood was correlated with cumulative ACE exposure and postpartum mood symptoms (*p* = 0.006).

**Conclusions:**

Trauma across the life course is associated with perinatal violence, infant removal, and postpartum mood symptoms among women on community supervision. Trauma-informed, gender-responsive perinatal and community supervision interventions are needed to improve safety, mental health, and family stability.

**Trial registration:**

NA

**Supplementary Information:**

The online version contains supplementary material available at https://doi.org/10.1186/s12884-026-09495-2.

## Background

Current evidence illustrates that childhood experiences of trauma such as adverse childhood experiences (ACEs) have detrimental health effects throughout the life course [8, 18, 72]. ACEs are typically defined as exposure to one or more of the following before age eighteen: experiencing emotional, physical, or sexual abuse; parents with substance use disorder or mental illness; incarceration of a family member; and parental separation or divorce [1, 18]. Exposure to childhood trauma may be indirectly associated with criminal legal system contact [52, 61]. Trauma exposure during adolescence significantly increases the risk of lifetime substance abuse, interpersonal violence, and mental health challenges, all of which increase the likelihood that an individual will experience incarceration [2, 19, 34].

Women in contact with the criminal legal system report disproportionately high rates of ACEs and continued trauma, which include domestic, intimate partner, and sexual violence into adulthood [37]. However, a smaller body of research investigating the health effects of ACEs and the continued trauma in the form of abuse (domestic, intimate partner, and sexual violence) during adulthood exists. This scholarship examines the intersection of incarceration and its effects on perinatal health (also known as the health of individuals who are pregnant, birthing, and postpartum) including outcomes such as violence during pregnancy, the permanent removal of one’s infant after childbirth, and postpartum mood symptoms [15].

In 2024, roughly 808,700 (73%) of the total amount of women within the criminal legal system were deferred to community supervision, a form of community based correction oversight, also known as probation, in the United States [33].

Community supervision is a broad category of criminal legal system surveillance, which includes probation, a community-based alternative to serving time in a prison or jail facility, and parole, when an individual finishes their court-ordered sentence within the community following time served in a carceral facility [21, 28]. Requirements of community supervision are strict and often include electronic monitoring, full-time education or employment, routine meetings with law enforcement officers, and community service [28].

Although community supervision provides an alternative to incarceration behind bars, strict requirements imposed by the court and law enforcement officers, in combination with a lack of structural support, can make community supervision a particularly vulnerable time. Individuals on community supervision report facing significant stigma seeking employment, stable housing, and treatment services; however, these actions are requirements to avoid violations and potential reincarceration [14, 50, 54]. Most of these women are of childbearing age, and many are experiencing issues related to perinatal health. Given that most women influenced by the criminal legal system are within childbearing age [21, 33], researchers must also strive to understand the effect of life course trauma on perinatal health outcomes for women on community supervision. Nevertheless, little research exists on the intersection of community supervision and trauma across the life course on perinatal outcomes.

A recent scoping review found only four studies evaluating perinatal outcomes among women on probation and parole, none of which centered on exposure to trauma [15]. The identified studies focused on mental health and substance use, finding that the parallel challenges of unmet health needs, lack of community support, and financial distress contributed to high rates of depression, as well as issues with substance use cessation and programming attrition [5, 58, 59, 64]. Addressing the gap in research around the impacts of lifelong trauma on perinatal outcomes during community supervision is essential to bridge access to care, enhance health equity, and reduce maternal morbidity and mortality during pregnancy, childbirth, and postpartum.

### Framework

**Fig. 1:**
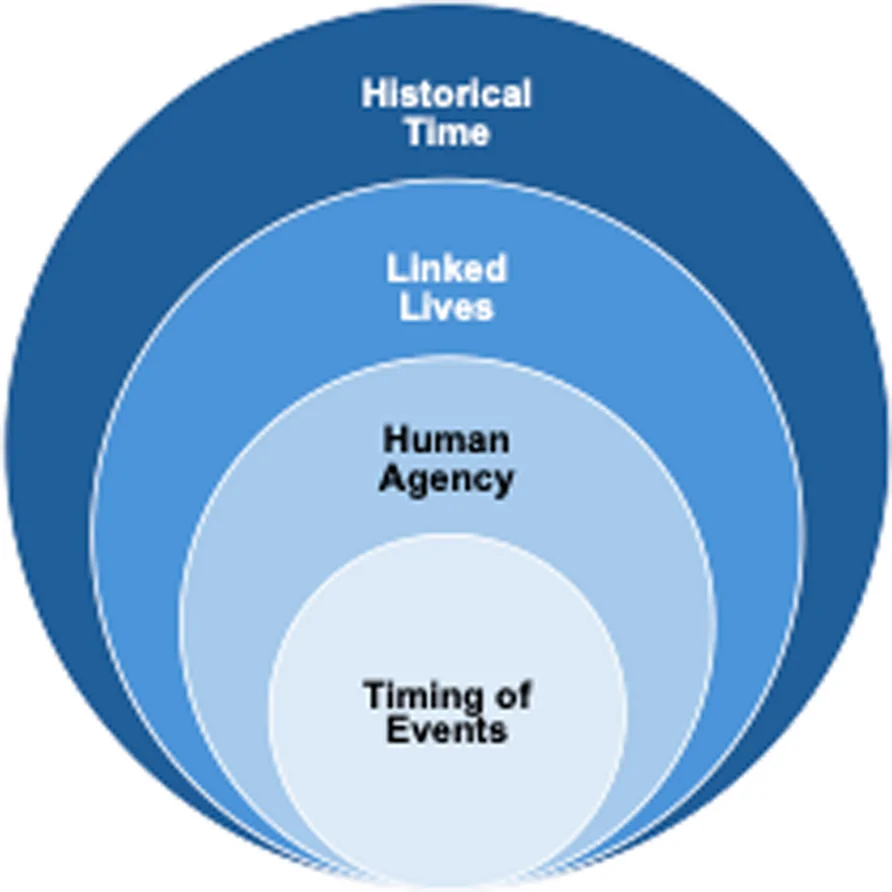
The basic principles of the life course theory

**Fig. 2:**
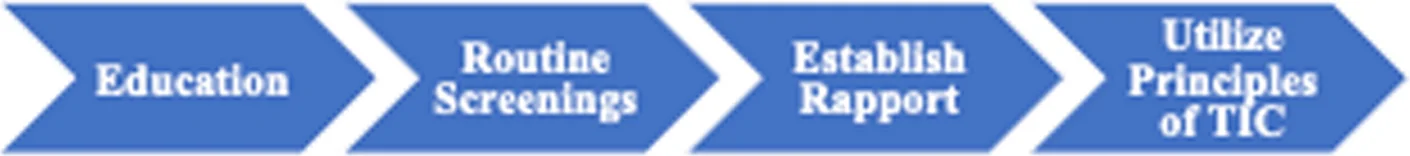
Steps nurses, healthcare providers, community workers, and officers can take to address trauma across the life course of those on community supervision during the perinatal period

This study is guided by Life Course Theory (LCT), a multidisciplinary framework that examines how social, structural, and individual experiences accumulate over time to shape health trajectories across the lifespan [17]. LCT posits that health is not the product of isolated events, but rather the result of interconnected experiences that unfold within specific contexts. Core principles of LCT include: (1) historical time and place, which emphasizes how broader social and institutional contexts shape individual lives; (2) linked lives, which recognizes that individuals’ trajectories are interdependent with those of family members, partners, and children; (3) human agency, referring to the capacity to make choices within structural constraints; and (4) timing of events, which highlights how the developmental timing of exposures (e.g., childhood trauma, pregnancy, incarceration) influences later outcomes [17] (Figs. 1 and 2).

Applied to women on community supervision, LCT provides a useful lens for understanding how early-life adversity (e.g., adverse childhood experiences [ACEs]) and later-life trauma (e.g., intimate partner, sexual, and domestic violence) accumulate and intersect with criminal legal system involvement to shape perinatal health outcomes. From an LCT perspective, pregnancy and the postpartum period represent sensitive life-course windows during which prior trauma, ongoing stressors, and structural constraints (e.g., surveillance, poverty, limited access to care) may exert particularly strong effects on mental health, safety, and family stability [60]. Using LCT, we therefore conceptualize perinatal outcomes not as isolated clinical events, but as the downstream expression of cumulative, interconnected exposures unfolding across childhood, adulthood, and the perinatal period. The goal of the current study was to examine trauma across the life course and the perinatal outcomes of women on community supervision.

## Methods

### Original study design

Our data were the result of a diversity supplement within an R01 (3R01HD103634-04S2 Principal Investigator [PI]: Allison D. Ihle) being conducted across eight different states and prison systems. As such, the parent study focused on the needs of women who were incarcerated, and our study used a similar focus to examine the needs and issues of women involved with the justice system and managed on community supervision. Following Institutional Review Board Approval (STUDY00000282), we used a cross-sectional design and the same survey tools used in the Parent R01(3R01HD103634-04S1) to measure participant demographics, ACEs, permanent removal of one’s infant after childbirth, and postpartum self-reported mood disorder symptoms to assess the perinatal outcomes of women on community supervision. For our study, we augmented the previously chosen surveys with others regarding abuse during pregnancy and across the life course to capture different types of violence (partner, sexual, perinatal) during adulthood. The decision to add a measure for perinatal and lifespan abuse was necessary since preliminary studies with this population highlighted instances of abuse within the community setting. To increase participant engagement with the research, we used a quantitative approach to ask survey questions in the form of a telephone interview to collect data specific to the participant’s most recent pregnancy while on community supervision to answer the following research question: What are the perinatal needs and outcomes of women on community supervision? This method of data collection worked well in prior studies of the PI.

### Current study design

The purpose of this secondary analysis was to examine the effects of trauma across the life course on the perinatal outcomes of women on community supervision. Research questions that guided data analysis included the following: (1) What are the correlations of ACEs on experiencing violence in pregnancy and postpartum?; (2) What is the correlation of trauma such as ACEs and abuse during adulthood (domestic, intimate partner, and sexual violence) on the separation of women and their children?; (3) What is the correlation of trauma such as ACEs and abuse during adulthood (domestic, intimate partner, and sexual violence) on postpartum mood symptoms?

We conducted a cross-sectional secondary analysis of data collected via structured telephone interviews with N = 60 women aged 18–50 years who experienced pregnancy, childbirth, and the postpartum period while under probation-based community supervision in South-Central Texas, USA. The parent study used standardized survey instruments to assess demographics, criminal legal history, and perinatal experiences. For this analysis, childhood trauma was measured using a shortened adverse childhood experiences (ACEs) scale adapted from Felitti et al., and trauma in adulthood and during pregnancy (domestic, intimate partner, and sexual violence) was assessed using the validated Abuse Assessment Screening tool. Postpartum mood symptoms were evaluated using the Patient Health Questionnaire-2 (PHQ-2) and the Generalized Anxiety Disorder-2 (GAD-2). Additional items assessed social support during childbirth and infant removal after delivery. The primary outcomes were perinatal violence, permanent infant removal after childbirth, and postpartum mood symptoms. Logistic regression models were used to examine associations between trauma exposures across the life course and perinatal outcomes. Given the ordinal nature of cumulative trauma measures, Kendall’s tau was used to estimate correlations between lifetime trauma and perinatal outcomes.

Our hypothesis was that high rates of ACEs and trauma across the life course such as domestic, intimate partner, and sexual violence in adulthood may correlate to higher rates of on perinatal violence, the permanent removal of one’s infant after childbirth, and postpartum self-reported mood disorder symptoms for women while on community supervision. To answer our research questions, a secondary analysis using logistic regression was completed to evaluate the association of trauma across the life course such as ACEs, and abuse during adulthood (domestic, intimate partner, and sexual violence) and its influence on perinatal violence, the permanent removal of one’s infant after childbirth, and postpartum self-reported mood disorder symptoms while on community supervision.

### Sample

Following Institutional Review Board Approval (IRB # 20230684H), we purposively sampled sixty individuals who self-identified as women who had been previously arrested and court-mandated to criminal legal oversight on community supervision department in South-Central Texas, a predominately Hispanic region within the United States of America. The community supervision department in which we recruited from serves a large metropolitan area with roughly 21,885 clients per month, with most between the age of 22–50 years (*n* = 17, 787; 81%); and a quarter self-identifying as women (*n* = 4,624; 25.6%) [3]. Inclusion criteria for participants were those who self-identify as female,18–50 years old; reside in Texas; and having experienced their most recent pregnancy, childbirth, and postpartum on court-mandated community supervision within the past five years. Exclusion criteria were those who could not write or read English, who did not identify as female, who were under the age of 18 years, and whose safety or mental health would be affected from participating.

With the help of the administrative team at the community supervision department, batch emails with the study flyer were sent to their clients. Participants were urged to call the PI to be screened for inclusion, a process that took approximately ten minutes. During this time, participants were read an information sheet and had opportunities to ask questions about the study to the PI. After enrollment in the study, participants were mailed a copy of the information sheet and a university-approved debit card. The university-approved debit card was then loaded with an incentive of sixty dollars at the conclusion of the data collection interview. The team could not reconnect with eleven participants to conduct data collection after enrollment and consent; therefore, we obtained IRB approval to consent more participants until we had met our target sample size of sixty. A total of 71 people were consented with 60 individuals completing data collection.

### Data collection

We collected data over the telephone and managed data with Research Electronic Data Capture (REDCap) [29] hosted at the University of Texas Health Science Center at San Antonio. We followed a safety protocol outlined by the IRB in case of any reports of self-harm and/or harming others (children and elderly) during data collection. The PI read the survey questions with the participants individually, which took approximately forty-five minutes to an hour. Survey variables extracted for analysis was guided by the LCT framework such the participant’s demographics, arrest and community supervision history and duration,and experiences of ACEs; quality of social support during childbirth; histories of trauma in adulthood (domestic, partner, and sexual violence); removal of infant after childbirth; and postpartum mood symptoms during the perinatal period (Supplemental Table 1).

### Measures

In the current study, internal consistency reliability could not be calculated for all measures due to the binary/ordinal nature of several variables (e.g., trauma exposure items and ACE categories). However, the use of validated instruments with established psychometric properties in prior research strengthens confidence in the measurement of key constructs.

#### Maternal demographic survey

The maternal demographic survey was adopted from the parent study (3R01HD103634-04S1and PI: Allison D. Ihle), has not been published elsewhere, and consisted of questions regarding participant’s age, race/ethnicity, education, and relevant health history specific to pregnancy. This survey also includes a shortened ACEs scale [18] consisting of five ACEs such as (i) having a caregiver who was incarcerated; (ii) experiences with abuse; (iii) the experiences of witnessing abuse; (iv) experiences of foster care; and (v) experiences of sexual abuse before the age of 18 years. The shortened adverse childhood experiences (ACEs) scale used in this study was adapted from the original 10-item ACEs measure developed by Felitti et al. [18]. While the full 10-item ACEs instrument has demonstrated good internal consistency in prior research (e.g., ω = 0.906) and is widely used to assess childhood trauma exposure among adult women, reliability indices specific to the five-item subset used here have not been previously published [45], internal consistency was acceptable for use in exploratory analyses in the current sample. We chose to focus on these five ACEs to decrease participant burden and expand off the parent study with the intent to conduct future analysis comparing our sample on community supervision to their sample consisting of women who are incarcerated (Supplemental Table 1).

We asked participants to report their ACEs before the age of 18 years and incarceration history related to the terms of their community supervision. We also asked participants about their satisfaction with the pregnancy support they received and their birth experience; mental health services prior to arrest and their current postpartum mood symptoms; and experiences with family separation. (e.g., “When do you expect to be off community supervision?”; “Did you live with anyone who went to jail or prison before the age of 18?”, “Was the infant permanently removed from you care after delivery?”) (Supplemental Table 1).

#### Social support and post-birth survey

Next, we assessed participants’ experiences with support during labor and postpartum, modifying measures used in our parent study (3R01HD103634-04S1and PI: Allison D. Ihle), (e.g., “How would you rate the care you received from your support person?; My support person advocated for me when I needed it; My personal preferences were respected and honored by my support person during labor and birth”) (Supplemental Table 1). Responses were summed and scored on a Likert scale: (Very Poor, Poor, Acceptable, Good, and Very Good). This tool has not been published elsewhere.

#### Patient health questionnaire and generalized anxiety disorder

The 2-item Patient Health Questionnaire (PHQ-2) and Generalized Anxiety Disorder (GAD-2) tool [56, 66] is a shortened valid and reliable tool used to measure depressed mood, anhedonia, and anxiety. This tool was also used in our parent study. The tools assessed symptom frequency and severity in the past two weeks (e.g., “Over the last two weeks, how often have you been bothered by the following problems…feeling down, depressed, or hopeless?”) (Supplemental Table 1). Responses were summed separately for the PHQ-2 and GAD-2 to yield a total score with clinically defined cut-points (e.g., cut-off scores > 3 were considered clinically elevated) [56]. The PHQ-2 (α = 0.83,ω = 0.80) and the GAD-2 (α = 0.74,ω = 0.70) showed internal consistency scores and good reliability. These brief measures have established psychometric properties in prior research, with the PHQ-2 demonstrating internal consistency estimates of α = 0.83 and ω = 0.80, and the GAD-2 showing α = 0.74 and ω = 0.70 [56, 66]. These reliability estimates support the use of the PHQ-2 and GAD-2 as valid, reliable screens for depressive and anxiety symptoms in perinatal and general adult populations.

#### Abuse assessment screening

The Abuse Assessment Screening tool (AAS) [55] was used to assess violence such as domestic, intimate partner, and sexual violence in adulthood and during pregnancy within the last year and across the lifespan in adulthood. The AAS was not utilized by the parent R01 study (3R01HD103634-04S1and PI: Allison D. Ihle). We added this measure to account for context-specific issues women may experience on community supervision while being economically dependent on abusive partners (e.g., “Since you were pregnant, have you been physically hurt by someone? Within the past year, has anyone forced you to have sex?”). Questions have yes/no answers,if a respondent answers “yes,” options to specify the perpetrator are given (e.g., “husband…. stranger… multiple”; Supplemental Table 1).

The AAS tool was employed to measure domestic, intimate partner, and sexual violence in adulthood and during pregnancy. In prior validation studies, the AAS has demonstrated high sensitivity (93–94%) and moderate to high specificity (55–99%) for identifying abuse during pregnancy [44]. The AAS has also been used effectively in international samples of women to detect violence across the lifespan, supporting its utility in diverse populations. This screening tool has been utilized in other studies amongst women in Iran [25] and in Nepal [41].

### Data analysis

All statistical analyses were performed using R [51]. Descriptive statistics were used to summarize participant demographics, criminal legal system history, trauma exposures, social support, and perinatal outcomes. Continuous variables, including age, number of prior arrests, and years on community supervision, were summarized using means and standard deviations (SD). Categorical variables, including race/ethnicity, sexual orientation, education, history of mental health diagnoses, exposure to adverse childhood experiences (ACEs), experiences of domestic, intimate partner, and sexual violence, receipt of social support during childbirth, infant removal after delivery, and clinically elevated postpartum mood symptoms, were summarized using frequencies and percentages. These descriptive statistics were used to characterize the sample and to provide context for subsequent regression and correlation analyses.

For trauma exposure, individual ACE items were reported as the proportion of participants endorsing each exposure, and a cumulative ACE score (range 0–5) was also described using frequencies and percentages for each score category. To study the effects of ACEs on experiencing violence during the perinatal period in Aim 2, we used five items from the ACEs scale that were evaluated in the parent R01 study. We first applied a generalized linear model using variables in Table 1 that asked participants if they had experienced a parent who had been incarcerated, being physically abused, witnessing abuse, experiencing foster care, and sexual abuse, before the age of 18 years as covariates, with abuse as the response variable.

**Table 1:** ACES variables for generalized linear model

Variable	Estimate	Std. Error	z value	Pr(>|z|)
(Intercept)	0.7516	0.6312	1.191	0.23
Parental Incarceration	0.3574	0.8008	0.446	0.66
Physical Abuse	1.1075	1.2530	0.884	0.38
Witnessing Abuse	1.5568	1.2255	1.270	0.20
Foster Care	−0.4475	1.0111	−0.443	0.66
Sexual Abuse	0.3712	0.8015	0.463	0.6

Different types of ACEs (parent who had been incarcerated, being physically abused, witnessing abuse, experiencing foster care, and sexual abuse) before the age of 18 years

Each item was coded 0 = No/1 = Yes. A cumulative ACE score was created by summing these items, yielding a total score ranging from 0 to 5, with higher scores indicating greater childhood trauma exposure. To further explore whether ACEs have a cumulative effect, we followed the approach of Chartier et al. [12] and created an aggregate measure by combining the adverse childhood experience variables into a single cumulative variable [12]. Specifically, we summed the values of five covariates — having a parent who was incarcerated, being physically abused, witnessing abuse, experiencing foster care, and sexual abuse before the age of 18 years — to capture the overall impact of these experiences on the response variable abuse. This cumulative ACE score was used in correlation analyses (Kendall’s tau) and regression models to represent overall childhood trauma burden and the associated *p*-value to test for statistical significance. To evaluate the correlations of ACEs on experiencing trauma (domestic, intimate partner, sexual violence) in adulthood and/or the perinatal period, we applied the logistic regression model with caregiver incarceration (*p* = 0.36) before the age of 18, physical abuse before age 18 (*p* = 0.34), witnessing abuse before the age of 18 (*p* = 0.20), foster care before the age of 18 (*p* = 0.72), and prior sexual abuse before the age of 18 (*p* = 0.64) as covariates, using abuse as the response variable.

To assess for adulthood and perinatal trauma using the AAS, items assessing domestic, intimate partner, and sexual violence in adulthood and during pregnancy were coded as binary variables (Yes/No). Individual indicators (e.g., any adulthood violence, sexual violence, perinatal violence) were used as predictors or outcomes in logistic regression models. A dichotomous “any trauma in adulthood/perinatal period” variable was also used in some analyses. Postpartum mood symptoms used PHQ-2 to screen for self-reported depression symptoms which consisted of two items summed to produce a total score ranging from 0 to 6.

The GAD-2 assessed for self-reported anxiety using two items summed to produce a total score ranging from 0 to 6. For descriptive and analytic purposes, scores > 3 were used as the standard clinical cut-off indicating possible major depressive disorder (PHQ-2) or generalized anxiety disorder (GAD-2). In regression analyses, mood symptoms were analyzed as binary outcomes (above vs. below cut-off). For perinatal outcomes that were binary variables, we coded as Yes/No and used them as dependent variables in logistic regression models for (i) perinatal violence (any violence during pregnancy/postpartum); (ii) permanent infant removal after childbirth; and (iii) clinically elevated postpartum mood symptoms (PHQ-2 > 3 and/or GAD-2 > 3).

We used Kendall’s tau to assess associations between the binary or ordinal perinatal outcomes (e.g., violence, infant removal, mood symptoms). This approach was chosen due to the ordinal and non-normally distributed nature of the trauma exposure variables. To measure the overall effects, we combined these five covariates into a single covariate, representing their total frequency: caregiver incarceration before age eighteen + physical abuse before age eighteen + witnessing abuse before the age of eighteen + foster care before the age of eighteen + prior sexual abuse before the age of eighteen. This approach allowed us to assess the collective impact of the five covariates on the response variable trauma. Due to the nature of the covariates under consideration, we used Kendall’s tau to estimate the ordinal association between the two measured quantities based on the ranks of the data. We then use its corresponding *p*-value to test for statistical significance.

## Results

Details regarding the demographic and health history data of the sample can be found in Table 2. We also discuss results specific to the quality of social support during childbirth and the correlations between ACEs and trauma (domestic, intimate partner, sexual) violence in adulthood on perinatal violence, the permanent removal of one’s infant after childbirth, and postpartum self-reported mood disorder symptoms.

**Table 2:** Participant demographic characteristics, N = 60

Age	n (%)
18–25	16 (27)
26–34	32 (53)
35–44	11 (18)
45–50	1 (2)
Race	n (%)
Black or African American	3 (5)
Multiple	3 (5)
Other	1 (2)
White	53 (88)
Hispanic/Latino	n (%)
	41 (68)
Employment^a^	n (%)
Not employed	27 (46)
Full time	15 (25)
Part time	16 (27)
Contract work	1 (2)
Where do you live?	n (%)
Home	25 (42)
Apartment	26 (43)
Group home	1 (2)
Recovery center	2 (3)
Homeless	6 (10)
Highest education level	n (%)
8th grade or less	3 (5)
Some high school	9 (15)
High school diploma/GED	23 (38)
Some college/Postsecondary	14 (23)
Associate degree	3 (5)
Trade apprenticeship, licensure, or other certification program	6 (10)
Bachelors degree	2 (3)
Relationship status	n (%)
Married	6 (10)
Single	32 (53)
Divorced	2 (3)
Separated	3 (5)
Open relationship	1 (2)
Living with partner	16 (27)
Prior times arrested	M (SD)
	4.1 (7)
Estimated time on probation (years)	M (SD)
	5.0 (3)

Missing values, where applicable, shown as superscripts^a^

### Demographic and health history data

The sample had been arrested on average four times before (*SD* = 7), with an average of five (*SD* = 3) years on community supervision. All the sample (*n* = 60, 100%) self-identified as female with most (*n* = 50, 83%) identifying as heterosexual, and Hispanic ethnicity (*n* = 41, 68%). Most participants (*n* = 52, 87%) had experienced partner violence, experiencing violence during their most recent pregnancy (*n* = 21, 35%), or (*n* = 15, 25%), and reported violence within the past year. Many participants had been diagnosed with mental illness in the past (*n* = 45, 75%), with current self-reported symptoms of depression (*n* = 40, 67%), anxiety (*n* = 31, 52%), post-traumatic stress (PTSD) (*n* = 13, 22%), and bipolar disorder (*n* = 13, 22%). Most participants (*n* = 55, 92%) stated they were not receiving mental healthcare services prior to their last arrest.

### ACEs

Across the sample when considering the ACEs, participants experienced the incarceration of their caregiver (*n* = 34, 56%), physical abuse (*n* = 25, 42%), witnessing abuse of others (*n* = 27, 45%), experiencing foster care (*n* = 17, 8%), or sexual abuse (*n* = 29, 48%) before the age of eighteen years. When it came to the total ACE score out of a shortened scale of five ACEs, participants (*n* = 9, 16%) in the sample reported four adverse experiences: being physically abused, witnessing physical abuse, experiencing foster care, and sexual abuse before the age of eighteen, while others experienced all five ACEs (*n* = 6, 10%).

### Social support during childbirth

The findings related to social support are retrospective self-accounts of participant’s perceived social support at the hospital during their most recent childbirth while on community supervision. Most participants (*n* = 49, 82%) had support in the hospital during labor and postpartum. Almost half the sample of participants (*n* = 29, 47%) expressed support from their partner, while others had support from their parent (*n* = 9, 15%), a friend (*n* = 7, 11%), or their sibling (*n* = 3, 5%) during labor. Out of the sample, most women (*n* = 55, 92%) stated they would have liked additional support such as a doula in future pregnancies. Many participants also expressed they couldn’t ask questions (*n* = 44, 73%) to medical staff during labor and delivery with many (*n* = 38, 63%) voicing that they were not advocated for during their birth experience.

### Correlations between ACEs and trauma in adulthood

The association between trauma across the life course such as ACEs and trauma in adulthood such as domestic, intimate partner, sexual violence and their influence on perinatal violence, the permanent removal of one’s infant after childbirth, and postpartum self-reported mood disorder symptoms will now be reported.

#### Perinatal violence

The results illustrate a correlation between ACEs on experiencing trauma (domestic, intimate partner, sexual) violence in the perinatal period with various responses and report the Kendall’s tau and its corresponding *p*-value in Table 3.

**Table 3:** Effects of ACES on Experiencing trauma in the perinatal period

Response variable	Kendall Tau	*P*-value
Abuse (yes/no)	0.2496417	0.03
Physical Abuse	0.1634114	0.16
Abuse during Pregnancy	0.1987179	0.09
Forced Sex	0.2392518	0.04

Abuse includes total amount of domestic, intimate partner, and sexual violence during pregnancy, within the past year, and across adulthood

There is a statistical correlation between history of violence in adulthood and overall impact of ACEs on experiencing violence during the perinatal period (*p* = 0.03). In addition, there was a correlation between forced to have sexual intercourse and overall impact of ACEs on experiencing violence during the perinatal period (*p* = 0.04).

#### Permanent removal of infants

To evaluate the effects of violence across the lifespan on the separation of women and their children in the postpartum period, we evaluated rates of permanent removal of infants from Child Protective Services (CPS) and rates of those who were discharged separately from their infant’s following childbirth. We found a correlation between violence in adulthood and the permanent removal of an infant by CPS following childbirth during the postpartum period (*p* = 0.003) (Table 4).

**Table 4:** Effects of violence on the separation of infants after childbirth while on community supervision

Response variable	Kendall Tau	*P*-value
Permanent Removal of Infant (1st)	−0.08338688	0.49
Discharged without Infant (2rd)	−0.2242795	0.14
Permanent removal of Infant (3rd)	−0.3566764	0.003
Discharged without Infant (4th)	−0.0256283	0.87

#### Self- reported postpartum mood symptoms

Finally, when we evaluated the effects of trauma across the life course on self-reported postpartum mood symptoms, we found a correlation between sexual violence, mood symptoms in adulthood, and ACEs on experiencing mood symptoms in the postpartum period (*p*-value = 0.006) (Table 5).

**Table 5:** Effects of trauma across the life course on mood symptoms on the perinatal period

Response variable	Kendall Tau	*P*-value
PHQ – 2 Total score	0.01549568	0.89
GAD – 2 Total score	−0.0225183	0.84
PHQ-2, GAD-2 combined scores	0.298202	0.009
PHQ-2, GAD2 combined scores	0.3313589	0.006

The 2-item Patient Health Questionnaire (PHQ-2) and Generalized Anxiety Disorder (GAD-2) tool [66] is a shortened valid and reliable tool used to measure depressed mood, anhedonia, and anxiety

The sample’s depression and anxiety self-reported scores two weeks prior to the interview resulted in participants (*n* = 20, 33%) with cut off scores of > 3 for the PHQ-2, which suggested possible major depressive disorder [56, 66]. Further, most participants (*n* = 34, 57%) had cut off scores of > 3 for the GAD-2 which suggest possible generalized anxiety disorder [56, 66].

## Discussion

In this secondary analysis, we examined how trauma across the life course is associated with perinatal outcomes among women on community supervision in South-Central Texas. Guided by LCT, our findings demonstrate that ACEs and trauma in adulthood—including domestic, intimate partner, and sexual violence—are significantly associated with perinatal violence, permanent infant separation, and postpartum mood symptoms. These results underscore that perinatal health among women under criminal legal supervision cannot be understood in isolation from earlier life experiences, ongoing social contexts, and structural constraints, but rather reflects the cumulative and interconnected effects of trauma over time [17, 27].

### Historical time and accumulation of risk

Consistent with the LCT principle of historical time, participants’ life trajectories were marked by early and repeated exposure to adversity, including caregiver incarceration, physical and sexual abuse, witnessing violence, and foster care involvement. More than half of the sample reported multiple ACEs, with a notable subset experiencing four or more. Prior research demonstrates a clear dose–response relationship between ACE exposure and long-term morbidity and mortality, including mental health disorders, substance use, and chronic disease [18, 24, 70]. Our findings extend this literature by showing that cumulative childhood adversity is also associated with heightened vulnerability to violence during the perinatal period and poorer postpartum mental health among women on community supervision.

These results align with large cohort studies showing that higher ACE exposure increases risk for adverse pregnancy outcomes such as gestational hypertension, preterm birth, and postpartum mood disorders [46, 60]. From a life course perspective, the perinatal period may represent a critical “stress-sensitive” window during which the biological and psychosocial consequences of cumulative trauma are amplified. The high prevalence of depressive and anxiety symptoms observed in this sample is consistent with evidence that childhood and lifetime trauma increase vulnerability to mood disorders across adulthood and into the postpartum period [1, 39]. Moreover, repeated arrests and prolonged periods on community supervision in this sample suggest that early and ongoing trauma may contribute to trajectories characterized by instability, untreated mental health needs, and continued criminal legal system involvement—patterns well documented in prior work on intergenerational trauma and justice involvement [6, 31].

### Linked lives, social context, and family disruption

The LCT principle of linked lives emphasizes that individual health trajectories are embedded within family and social networks [17]. In this study, participants’ experiences of pregnancy, childbirth, and postpartum were deeply shaped by their relationships with partners, family members, and children, as well as by the constraints of community supervision. Although most participants reported having a support person during childbirth, many described inadequate advocacy, limited ability to ask questions, and a desire for additional support such as doulas.

At the same time, our findings revealed a strong association between violence in adulthood and permanent infant removal, highlighting how trauma, surveillance, and family separation are tightly intertwined. Prior scholarship has shown that women involved in the criminal legal system face elevated risks of family disruption, child welfare involvement, and long-term intergenerational harm [7, 62, 69]. From a life course perspective, these disruptions not only affect mothers’ health and well-being, but also shape the developmental trajectories of their children, reinforcing cycles of adversity and system involvement across generations [6, 31].

### Human agency under structural constraint

The principle of human agency highlights individuals’ capacity to make choices, while recognizing that these choices are constrained by social, economic, and institutional contexts [17]. In this sample, high rates of recent and lifetime violence, limited education, unstable housing, and economic precarity suggest that women’s ability to exercise agency—such as leaving abusive relationships or consistently accessing healthcare—was severely constrained. These findings are consistent with prior research showing that women under criminal legal supervision face significant barriers to employment, education, and safety, particularly when navigating abusive relationships [16, 23, 34]. Within the perinatal period, these constraints may be especially consequential. Limited agency in the context of pregnancy and postpartum recovery can increase exposure to violence, worsen mental health, and reduce access to timely and appropriate care, thereby compounding already elevated risks for morbidity and mortality.

### Timing of events and perinatal vulnerability

Finally, the LCT principle of timing of events underscores the importance of when exposures occur in the life course [17]. The overlap of pregnancy, postpartum recovery, caregiving responsibilities, and community supervision represents a particularly high-risk convergence of stressors. Most women under community supervision are of childbearing age, and many are primary caregivers [9, 11, 42]. Our findings that women remain under supervision for several years during these life stages suggest prolonged exposure to surveillance, financial strain, and time constraints during critical periods for both maternal and child health.

Prior research indicates that these pressures increase the risk of technical violations, reincarceration, and worsening health outcomes [54]. From a life course perspective, this convergence of stressors during sensitive developmental periods for both mother and child may have lasting implications for health, family stability, and intergenerational equity.

## Implications for practice and policy

Taken together, these findings highlight the need for trauma-informed, life course–oriented approaches within both healthcare and community supervision systems. First, routine screening for ACEs, lifetime trauma, and current violence during pregnancy and postpartum should be implemented using trauma-informed care principles to avoid re-traumatization and to facilitate timely referral to supportive services [13, 38, 65].Training for healthcare providers, probation and parole officers, and allied professionals is essential, as knowledge gaps in trauma-informed screening and response remain common [43, 49].

Second, interdisciplinary partnerships are critical. Integrated referral networks linking obstetric care, mental health services, domestic violence advocacy, substance use treatment, and community-based supports can help address the complex, intersecting needs of this population [30, 67, 67, 68, 68]. Community-based perinatal supports, including doulas and community health workers, have demonstrated effectiveness in improving birth experiences, mental health outcomes, and access to care, particularly among structurally marginalized populations [26, 40, 57].

Third, adaptive and technology-enabled interventions, such as mobile health (mHealth) and telehealth platforms, offer promising, low-barrier strategies to support symptom monitoring, safety planning, and care coordination among women with limited access to traditional healthcare [4, 10, 20, 35]. These tools may be especially valuable for individuals navigating the logistical and financial constraints of community supervision. Finally, policy-level interventions addressing the social determinants of health are essential. Access to stable housing, childcare, transportation, legal advocacy, and employment support can reduce dependence on abusive partners and improve both maternal and child outcomes [48, 53]. Community supervision agencies should consider perinatal accommodations, such as virtual appointments, reduced financial obligations, and flexible reporting requirements during pregnancy and postpartum, to mitigate unnecessary stress and health risks [47, 63, 71].

### Limitations

This study has several limitations. We sampled from one geographical region of South-Central Texas which is predominately Hispanic, potentially limiting generalizability of the findings. Despite this limitation, the characteristics of the sample adds to the literature by giving context to perinatal health issues of a racial/ethnic group that has been historically limited in research. Another limitation was the reliance on retrospective self-reported data which introduces the possibility of recall bias, particularly for lifetime trauma and perinatal experiences that may be difficult to accurately reconstruct. Studies using a longitudinal design and capturing data in real-time could address this limitation and is imperative in future research.

We also did not ask all the questions within the ACES survey but rather only focused on five ACEs, therefore, the total ACE score of the sample could be more significant had we included the other variables as part of the measure. In addition, we could not sample women managed through specialty courts that specifically dealt with those who were homeless, had a substance use disorder, or were arrested for sex-related offenses such as prostitution. Often those managed through specialty courts have been identified to have more severe forms of health disparities, barriers and risks to morbidity and mortality [22, 32, 36, 64]; therefore, our findings cannot be considered as a comprehensive representation of all pregnant, birthing, and postpartum individuals on community supervision.

Finally, the use of telephone interviews conducted by the principal investigator may also contribute to social desirability bias, potentially influencing how participants report sensitive or stigmatized experiences. The absence of objective verification of perinatal outcomes, such as confirmation through medical records, limits the ability to validate the self-reported data. The small sample size also reduces statistical power, especially for regression models, and may contribute to unstable estimates. Likewise, with cross-sectional design, no temporal or causal inferences can be drawn regarding the relationships among trauma, psychosocial factors, and perinatal outcomes. However, these limitations were unavoidable given the small timeframe (two years) and limited funding within our award to make following a larger sample longitudinally unfeasible. More research focused on these groups is needed.

## Conclusions

Our findings demonstrate that trauma across the life course can lead to adverse perinatal outcomes in women on community supervision. More research must focus on gender-specific issues that address women’s trauma leading to community supervision. Community-based interventions that utilize holistic methods of care such as doulas, community health workers, and technology could alleviate health disparities during the perinatal period for women on community supervision. Finally, community supervision departments and systems serving this population of women must consider the needs of individuals during pregnancy, childbirth, and postpartum. Steps to make accommodations to the perinatal needs of women may lead to better health outcomes and reduce recidivism.

## Supplementary Information

Supplementary Material 1.

## Data Availability

The datasets used and/or analyzed during the current study are available from the corresponding author on reasonable request.

## Abbreviations

ACEs: Adverse childhood experienced
LCT: Life Course Theory
PI: Principal Investigator
CPS: Child protective services
IRB: Institutional Review Board Approval
REDCap: Research Electronic Data Capture
PHQ-2: Two-item Patient Health Questionnaire
GAD-2: Two-item Generalized Anxiety Disorder Questionnaire
AAS: Abuse Assessment Screening tool
PTSD: Post-traumatic stress disorder
