# Supplementary material for: Correlations between life course trauma on perinatal outcomes of women on community supervision – a retrospective cross sectional study
Source: BMC Pregnancy Childbirth. 2026 Sep 3;26:994. doi: 10.1186/s12884-026-09495-2 (PMC13543587; doi:10.1186/s12884-026-09495-2)
Supplement: Supplementary file 1 [file 12884_2026_9495_MOESM1_ESM.docx]

**Supplemental Table 2**

**Supplemental Table 2A:** *Demographic and Pregnancy History Survey*

Demographic Survey Participants	diversity women

**DEMOGRAPHIC INFORMATION**

1. How old are you?	18-25

26-34

35-44

45-50

2. How would you describe your race? (SELECT ALL THAT American Indian or Alaska Native Tribal APPLY)	Affiliation:

 Asian

 Black or African American

 Native Hawaiian or Other Pacific Islander White

 Other:

2a. Do you identify as Latino/a/x? Yes No

3. How do you identify your gender?	Female

Non-Binary/Gender Non-conforming Male

Other:

3. What is your sexuality?	a. Heterosexual

b. Gay
c. lesbian
d. Bisexual
e. Pansexual
f. Asexual
g. other

4. What is your employment status?	a. Not employed

b. Full time
c. Part time
d. Contract work

5. Where do you live?	a. Home

b. Apartment
c. Group home
d. Recovery center
e. Homeless

6. What is the highest level of education you	8th grade or less

completed?	Some high school

High school diploma/GED Some college/Postsecondary Associate degree

Trade apprenticeship, licensure, or other certification program

Bachelor's degree Master's degree Doctoral degree

What is your relationship status?	a. Married

b. Single
c. Divorced
d. Separated
e. Open relationship
f. Living with partner

Are you in a monogamous relationship?	Yes No

a. If no, how many sexual partners do you have on average at one time?
b. If yes, how long have you been in your relationship?

5. In what county did you reside prior to this arrest/probation?

County State

|  | 6. What date did you get put on probation? |  | |
| --- | --- | --- | --- |
|  |  |  |  |
|  | 7. Approximately when do you expect to be done with probation? |  |  |
|  |  |  |  |
|  | 8. Are you on probation now for a new charge or a technical violation? |  New Charge |  Technical Violation |
|  | 8a. How long were you in jail before being on probation? |  |  |
|  |  |  |  |
|  | 9. Have you ever been in prison before this? (Please do not count jail time before being transferred to prison for this current offense) |  Yes  No | NR  |
|  | 9a. Approximately how many times have you been in prison prior to this arrest? |  |  |
|  |  | (Total #) |  |
|  | 9. Have you ever been in jail before this? (Please do not count prison time before being |  |  |
|  | transferred to prison for this current offense) |  |  |
|  | How many times have you been to jail before this last arrest? |  |  |

| What was the nature of your prior arrests? | traffic ticket technical violation misdemeanor felony  drug related offense | |
| --- | --- | --- |
|  | theft DWI/DUI | assault prostitution |
| How many times for each type of offense? |  |  |
| 1, traffic ticket |  |  |
| 2, technical violation  3, misdemeanor  4, felony  5, drug related offense 6, theft  7, assault  8, prostitution  9, DWI/DUI |  |  |
| **Before the age of 18...** |  |  |
| 10. Did you live with anyone who went to jail or prison? |  Yes  | No |
| 11. Did a parent or adult in your home ever physically hurt you? |  Yes  | No |
| 12. Did your parents or adults in your home ever harm or threaten to harm each other? |  Yes  | No |
| 13. Did you ever spend time in foster care? |  Yes  | No |
| 14. Did you ever experience unwanted sexual contact? |  Yes  | No |
| **Since you turned 18...** |  |  |
| 15. Have you ever experienced unwanted sexual contact? |  Yes  | No |
| 16. Have you ever experienced domestic violence? |  Yes  | No |
| Did you ever report the assault? | Yes No  |  |
| If you never reported the assault, can you share why (if you feel comfortable). |  |  |

Client Intake Interview Version	v2.080822 v3.031523 v4.072723

**PREGNANCY AND BIRTH HISTORY**

PREGNANCY AND BIRTH HISTORY Date Completed:

1. When was your baby's due? Date:

Was this a planned pregnancy?	Yes

No

If this was not a planned pregnancy, was it due to: unwanted sex/rape not using protection

 protection defected (ripped condom, missed pill) not having access to protection or birth control no having access to abortion

 other

If you feel comfortable explaining, can you please give more details?

IF CLIENT IS POSTPARTUM AT INTAKE

2. Who is caring for your baby now? (e.g., baby's other parent, maternal grandmother, foster family, etc.)

2a. How long has your baby been in this person's care?

 Weeks Months

Why does this person care for your child(ren)?

2b. Has there been a change in your baby's caregiver since birth?

 Yes No No Answer

2bi. Please explain:

3. Did you have any postpartum check-ups after giving birth?

Yes No IDK No Answer

3c. Were any concerns identified at your first postpartum check-up?

Yes No IDK No Answer

3d. Please describe any concerns identified at your first postpartum check-up:

4. Before this [current / most recent] pregnancy, how many times have you been pregnant?

**PREGNANCY HISTORY**

4a. Would you be comfortable discussing your prior pregnancies?

Yes No IDK No Answer

5. Have you ever been pregnant while in prison or jail?

Yes prison	No prison

yes jail	no jail	No Answer

5a. Can you tell me a bit about that (those) experience(s)

6. Have you ever given birth while in prison or jail?	Yes prison	No prison

yes jail	no jail	No Answer

6a. Where did your baby go after birth? (e.g., baby's father, maternal grandmother, foster family)

7. Based on your pregnancy history, how many pregnancies have resulted in live births?

(Total #)

7a. If you are comfortable sharing, would you please describe the outcomes of your other pregnancies?

8. How many of your births have been: Vaginal?	Cesarean?

VBACs?

9. Have you ever experienced any pregnancy complications?

(definition: any time during pregnancy, before onset of labor)

Yes	No	No Answer

9a. What pregnancy complications have you experienced?	Diabetes	(Select all that apply)	Other (Specify)

Other Pregnancy Complication

10. Have you ever experienced fetal complications? (definition: any time during pregnancy, labor, and birth until umbilical cord is cut)

Yes	No	No Answer

10a. What fetal complications have you experienced?

11. Have you ever experienced any labor or birth complications?

(definition: from the onset of labor through discharge from the hospital)

Yes	No	No Answer

11a. What complications have you experienced during labor or birth? (Select all that apply)

High Blood Pressure / preeclampsia:

Excessive bleeding that required treatment or blood transfusion:	Preterm birth:

Unplanned cesarean birth (not scheduled ahead of time) Shoulder dystocia

Other (Specify)

Other Labor or Birth Complications

12. Have your newborns ever experienced any complications?

(definition: after umbilical cord is cut and before discharge from the hospital)

Yes	No	No Answer

12a. What complications have your newborns experienced? (Select all that apply)

Spent time in the NICU: Neonatal Abstinence Syndrome (NAS) Other (Specify)

Other Complications

13. Have you ever experienced postpartum complications?

(definition: after discharge from the hospital through one year after birth)

Yes	No	No Answer

13a. What postpartum complications have you experienced? (Select all that apply)

Excessive bleeding that required treatment or blood transfusion:	High blood pressure:

Anything requiring hospitalization (e.g., infection or other post-birth complication) Mastitis, breast complications

Other (Specify)

Other Post Partum Complications

14. During previous postpartum experiences, were you ever diagnosed with a mood disorder?

Yes	No	No Answer

14a. Please provide more detail:

Postpartum Depression Postpartum Psychosis Other (Specify)

Other Mood Disorder

15. Was this [current / recent] pregnancy planned?	Yes	No	No Answer
16. How did you find out you were pregnant this most recent time?

Over the counter pregnancy test

During medical visit in the community During jail intake screening

During prison intake screening

5. During or after being on probation OTHER (SPECIFY):

If Other, please specify:

17. How far along were you when you found out you were pregnant?
18. What were your thoughts and feelings when you first learned you were pregnant?
19. How has community supervision impacted your plans and/or decisions for [this pregnancy / parenting your youngest child]?
20. How far along were you when you first started receiving prenatal care?

10. How many times do you meet a week with your probation officer?

How long do the requirements of your probation take per month?

how many hours per month do you probation requirements take?

What are the costs of your probation (restitution, urinalysis, etc.)?

Where do your children go while you check in with your probation officer?

Have you experienced intimate partner violence or	Yes

sexual violence since being on probation?	No

If you do not mind explaining, can you give me details of the violence you experienced?

26. Are you receiving additional food / nutrition?	Yes	No	No Answer

26a. Please describe

28. Are there any concerns with this postpartum recovery?

Yes	No	No Answer

28a. Please explain

29. What do you do now to feel connected to your baby?

PHYSICAL HEALTH HISTORY Date Completed:

**PHYSICAL HEALTH HISTORY**

30. How would you describe your general health? (circle one) Poor	Fair	Good	Very Good	Excellent

| 31. Do you have health insurance? | Yes | No | No Answer |
| --- | --- | --- | --- |
| 32. Do you have a routine check-up with a doctor in the community?  (Do not count prenatal care in the community) | Yes | No | No Answer |
| 33. Have you ever been told by a professional that you have a chronic medical condition?  (Do not include pregnancy-related concerns) | Yes | No | No Answer |
| 33a. Please describe: |  |  |  |

MENTAL HEALTH HISTORY Date Completed:

**MENTAL HEALTH HISTORY**

34. Have you ever been diagnosed with a mental illness?

Yes	No	No Answer

34a. What mental illness(es) have you been told you	Depression have? (SELECT ALL THAT APPLY):	Anxiety

Post-Traumatic Stress Disorder Bipolar Disorder

Personality Disorder Schizophrenia

Other:

35. Are you currently receiving services for a mental health concern?

Yes	No	No Answer

35a. What mental health services are you receiving?	Seeing a therapist or counselor on your own (SELECT ALL THAT APPLY)	Attending group therapy sessions

Seeing a psychiatrist for medications Other:

36. In the year before you were incarcerated, did you receive services for a mental health concern?

Yes	No	No Answer

36a. What mental health services did you receive?	Saw a therapist or counselor on your own (SELECT ALL THAT APPLY)	Attended group therapy sessions

Saw a psychiatrist for medications

Attended an intensive outpatient program Attended an intensive inpatient program Visited an emergency department in crisis Other:

SUBSTANCE USE HISTORY Date Completed:

**SUBSTANCE USE HISTORY**

37. Have you ever been diagnosed with a substance use disorder?

Yes	No	No Answer

37a. What treatment did you receive or was recommended you receive for your substance use disorder?

| 38. Are you currently receiving treatment for a substance use disorder? | Yes | No | No Answer |
| --- | --- | --- | --- |
| **MEDICATION HISTORY** |  |  |  |
| MEDICATION HISTORY Date Completed: |  |  |  |
|  |  |  |  |
| 39. Are you currently taking any medications, including prenatal vitamins, for any reason? This includes prescription or over-the-counter. | Yes | No | No Answer |

39a. What medications are you currently taking and what are they for?

40. When [you first learned] you were pregnant, did you start, stop, or make any changes to your medications?

Yes	No	No Answer

40a. Please describe:

HEALTHCARE EXPERIENCE IN PRISON Date Completed:

**HEALTHCARE EXPERIENCE While on Probation**

41. Have you received medical care while on community supervision for any concerns NOT related to pregnancy or postpartum?

Yes	No	No Answer

41a. Please rate the quality of the care you received.

Poor	Fair	Good	Very Good	Excellent

**Supplemental Table 2B:** *PHQ2-GAD2*

*Page 1*

1) PHQ2-GAD2 Version	v1.080222
2) Date:
3) 1. Over the last 2 weeks, how often have you been bothered by little interest or pleasure in doing things?

Not at all	Several Days	More than half the

days

Nearly every day

2. Over the last 2 weeks, how often have you been bothered by feeling down, depressed, or hopeless?

3. Over the last 2 weeks, how often have you felt nervous, anxious, or on edge?

4. Over the last 2 weeks, how often have you not been able to stop or control worrying?

**Supplemental Table 2C:** *AAS*

Abuse Assessment Screen Instructions: Circle Yes or No	Yes for each question	No

1Have you ever been emotionally or physically abused by your partner or someone important to you?

2. Within the last year, have you been hit, slapped,	Yes kicked or otherwise physically hurt by someone?	No

1) If YES, who? (Circle all that apply)	a. Husband
   b. Ex-Husband
   c. Boyfriend
   d. Stranger
   e. Other
   f. Multiple
2) If yes, how many times have you experienced this type of abuse?
3) 3. Since you've been pregnant, have you been slapped,	Yes kicked or otherwise physically hurt by someone?	No

4) If YES, who? (Circle all that apply)	a. Husband
   b. Ex-Husband
   c. Boyfriend
   d. Stranger
   e. Other
   f. Multiple
5) If yes, how often were you physically abused while pregnant?

Mark the area of injury on the body map.

Score each incident according to the following scale: SCORE

1 = Threats of abuse including use of weapon

2 = Slapping, pushing; no injuries and/or lasting pain

3 = Punching, kicking, bruises, cuts, and/or continuing pain

4 = Beating up, severe contusions, burns, broken bones

5 = Head injury, internal injury, permanent injury

6 = Use of weapon; wound from weapon	[Attachment: "body.jpeg"]

6) What is the total number/score for the times you have had abuse using the score below:

1 = Threats of abuse including use of weapon

2 = Slapping, pushing; no injuries and/or lasting pain

3 = Punching, kicking, bruises, cuts, and/or continuing pain

4 = Beating up, severe contusions, burns, broken bones

5 = Head injury, internal injury, permanent injury

6 = Use of weapon; wound from weapon

7) Within the last year, has anyone forced you to have	Yes sexual activities? YES NO	No

8) a. Husband

If YES, who? (Circle all that apply)	b. Ex-Husband

c. Boyfriend
d. Stranger
e. Other
f. Multiple
9) How many times total have you been forced?
10) 6. Are you afraid of your partner or anyone you listed	Yes above?	No

**Supplemental Table 2D:** *Social Support and Post-Birth Survey*

Survey Date

Very dissatisfied	Dissatisfied	Neutral	Satisfied	Very satisfied

1. Overall, how satisfied are you with your birth experience?
2. Overall, how satisfied were you with the prenatal care and support you received from the doctors and nurses in the clinic??
3. Overall, how satisfied were you with the role of the probation staff when it was time to go to the hospital?
4. Overall, how satisfied were you with the role of the officers who were seeing when it was time for you to go to the hospital to give birth?
5. Overall, how satisfied were you with the care you received from the hospital staff during your birth?
6. Overall, how satisfied were you with the cleanliness of the hospital where you gave birth?
7. Overall, how satisfied were you with the options you were offered at the hospital? (i.e., pain management, mode of delivery, etc.)
8. Overall, how satisfied were you with the time you spent with your baby after the birth?
9. Did you participate in prenatal and postpartum groups in prison?

Yes	No

**IF YOU PARTICIPATED IN PRENATAL AND/OR POSTPARTUM GROUPS:**

9a. How satisfied are you with the prenatal education and support you received from the prenatal and postpartum groups in the community while on supervision?

Very dissatisfied	Dissatisfied	Neutral	Satisfied	Very satisfied

10. Did you feel the prenatal and postpartum groups in the community provided an opportunity for you to get support from other participants?

Yes	No

IF YOU FELT A SENSE OF COMMUNITY IN PRENATAL AND/OR POSTPARTUM GROUPS:

10a. How satisfied are you with the support you received from other participants in prenatal and postpartum groups in the community?

Very dissatisfied	Dissatisfied	Neutral	Satisfied	Very satisfied

11. I was treated poorly by hospital staff because of my incarceration history or probation status?.

Strongly

disagree

Disagree	Neutral	Agree	Strongly agree

12. Please tell us a bit more about your experiences with community supervision staff during your pregnancy, labor, birth, and postpartum periods.
13. Please tell us a bit more about your experiences with hospital staff during your pregnancy, labor, birth, and postpartum periods.
14. Since you gave birth, how many times have you been able to visit with your baby?

(TOTAL #)

14a. If you were able to visit with your baby, where did you visit?

(LOCATION)

14b. If you wanted to visit with your baby but were unable to, what were the barriers?

15. Since you gave birth, how many times have you been able to contact your baby's caregiver?

Never	Once a Month	Once a Week	More Than Once a Week	Daily

15a. How were you able to contact your baby's caregiver (e.g., phone, in person, video call [e.g., Zoom], etc.)?

16. What was the best thing about the care you and/or your baby received while you were in the hospital? Please provide as much detail as you feel comfortable sharing.
17. What is one thing you wish had been different about the care you and/or your baby received while you were in the hospital? Please provide as much detail as you feel comfortable sharing.
18. What additional comments do you have about your labor and birth experience?
19. What comments do you have about the time you spent with your baby in the hospital after birth?
20. What comments do you have about your postpartum experience in general at the hospital?
21. What comments do you have about your postpartum experience if you experienced rearrest?
22. Have you seen or do you have an appointment to see a doctor for a standard postpartum checkup?

Yes	No

22a. What date did you have your postpartum checkup or is it scheduled?

22b. Overall, how satisfied awere you with the quality of the care you received at your postpartum checkup?

Very dissatisfied

Dissatisfied	Neutral	Satisfied	Very satisfied	My

appointment hasn't occurred yet

23. Please share any more comments you have about your postpartum care.
24. Was a doula or suppoer person present at any point during your hospital stay?

Yes	No

What type of support did you have?	doula	support person (partner/spouse) support person family

(aunt/uncle/cousin/sibling support person child support person friend other

Explain who supported you

| 25. How would you rate the care you received from your support person? | Very poor | Poor | Acceptable | Good | Very good |
| --- | --- | --- | --- | --- | --- |
|  | Strongly | Disagree | Neutral | Agree | Strongly agree |
|  | disagree |  | | | |
| 26. My support person advocated for me when I needed it |  |  |  |  |  |
| 27. My personal preferences were respected and honored by my support person during labor and birth. |  |  |  |  |  |
| 28. I felt comfortable asking my support person questions during labor and birth. |  |  |  |  |  |
| 29. If I am pregnant and placed in probation again in the future, I would want a support person there like a doula or community health worker. |  |  |  |  |  |

31. Please tell us a bit more about your experiences with your support person during your pregnancy, labor, birth, and postpartum periods.
